# Bilateral Distal Patellar Tendon Rupture in a 10‐Year‐Old Child: A Case Report and Review of the Literature

**DOI:** 10.1155/cro/3635857

**Published:** 2026-05-29

**Authors:** Elvin Gurbanov, Oscar Vazquez, Tiago Guedes Almeida, Dimitri Ceroni, Giacomo De Marco

**Affiliations:** ^1^ Pediatric Orthopedics Unit, Pediatric Surgery Service, Geneva University Hospitals, Geneva, Switzerland, hug-ge.ch; ^2^ Faculty of Medicine, University of Geneva, Geneva, Switzerland, unige.ch

**Keywords:** bilateral, patella, pediatric, rupture, tendon

## Abstract

**Background:**

Patellar tendon rupture is a rare lesion that usually occurs in adult patients with predisposing factors. The condition is even rarer in pediatric populations and presents either as a sleeve fracture of the patella’s inferior pole or as an avulsion of the tibial tubercle. The present report describes the case of a healthy 10‐year‐old female with bilateral, purely tendinous distal avulsions of her patellar tendons and considers her clinical presentation, treatment, and the clinical outcome.

**Methods:**

A 10‐year‐old female was admitted to Geneva University Hospitals and diagnosed with bilateral patellar tendon ruptures. The patient underwent bilateral tendon repair using four anchor sutures for each tendon and augmentation with a transpatellar metallic wire.

**Results:**

Postsurgical follow‐up out to 1 year showed good results, with the restoration of a full range of motion, an absence of pain, and a complete return to sports activities.

**Conclusion:**

This case report describes the very rare bilateral rupture of an otherwise healthy 10‐year‐old female’s patellar tendons in their soft tissue substance. It details the treatment, follow‐up, and positive outcome.

## 1. Introduction

Patellar tendon avulsion is a rare but highly debilitating condition in adults. The general principles of patellar tendon rupture, including mechanism, presentation, and management, have been well described in the orthopedic literature [1]. The rupture often occurs after an eccentric contraction of the knee’s extensor mechanism, resulting in a break in its continuity [2]. Conditions predisposing individuals to a tendon rupture include local or systemic corticosteroid therapies and prior acute or overuse injuries, such as chronic tendon pathologies and inflammation [3]. Patellar tendon avulsion primarily affects young males in their 20s or 30s [4].

Diagnosing a patellar tendon rupture can be challenging. When the rupture occurs, patients present with a lack of knee extension, swelling, and pain over the tibial tubercle or at the patella’s inferior pole [3]. Moreover, a gap between the tibial tubercle and the patella’s inferior pole can often be seen and palpated. Depending on the extent of the tear, ambulation may still be possible with the knee held in a slightly flexed position.

Anteroposterior and lateral X‐ray images usually reveal a patella alta caused by a superior migration of the patella and resulting in a higher Insall–Salvati ratio [5]. Conventional X‐ray images may also reveal an avulsion fracture of the patella’s distal pole (a sleeve fracture) or of the tibial tubercle. In expert hands, the tear and its extension can be evaluated using ultrasound. However, magnetic resonance imaging (MRI), when available, remains a very useful examination for precisely identifying the tear’s location and extent and for detecting any associated injuries, such as anterior cruciate ligament (ACL) or meniscal ruptures [6].

Patellar tendon rupture in pediatric populations is even rarer than in adult populations. Tendinous strength is greater in this age group, which may be why apophyseal avulsion fractures—like a tibial tubercle fracture or a sleeve fracture of the patella’s inferior pole—are more common than a rupture of the tendon substance itself [7]. The present report describes the case of a healthy 10‐year‐old female with bilateral, purely tendinous distal avulsions of her patellar tendons and considers the clinical presentation, treatment, and positive outcome.

## 2. Case Description

A healthy 10‐year‐old female was admitted to our university hospital emergency department after a gymnastics accident. She complained of bilateral knee pain and an inability to bear weight after a fall on her knees while landing from a jump. The patient had no history of any previous diseases or inflammatory tendon pathologies prior to her accident. Clinical examination of the left knee showed skin bruises and significant swelling over the tibial tubercle, associated with tenderness and pain at the distal patellar tendon insertion. Moreover, she was unable to perform a straight‐leg raise due to a deficiency of the extensor mechanism. The right knee had no bruises or swelling but was tender at the tibial tubercle site. Its extensor mechanism was also deficient.

**Figure 1:**
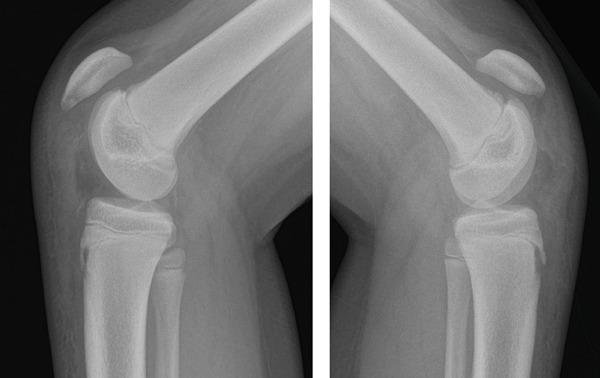
Right and left knee lateral X‐ray images showing patella alta and subcutaneous swelling in front of the tibial tubercle.

**Figure 2:**
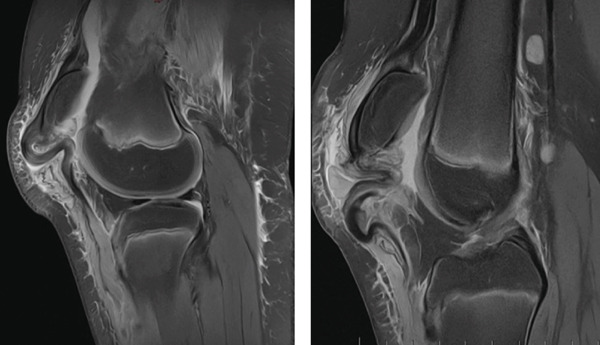
Right and left knee MRI showing purely tendinous distal patellar tendon avulsion.

Lateral X‐ray images of both knees revealed complete bilateral distal patellar tendon rupture with the proximal migration of both patellas (Insall–Salvati ratios: 1.9 left, 1.5 right). No fracture or avulsion was noted at either the distal patellar pole or at the level of the tibial tubercle. Soft tissue swelling was visible on both knees in front of the tibial tubercle, but without any intra‐articular joint effusion (Figure 1). MRI of both knees, obtained 48 h after the accident, showed bilateral distal avulsion of the patellar tendons with no other associated injuries (Figure 2). The patient’s parents gave their consent for bilateral open patellar tendon repair.

**Figure 3:**
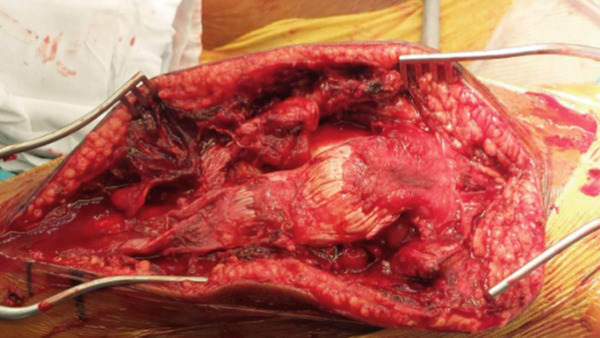
Intraoperative image of the left knee’s complete tendon avulsion.

A 15 cm midline incision was made in both knees, from the inferior pole of the patella down to the tibial tubercle. After dissection of the subcutaneous tissue and debridement of the hematomas, the distal tears were identified. The tendons were completely detached from the tibial tubercles (Figure 3). On the right side, the tendon was still in contact with the bone but presented with a consistent hematoma and a loss of tension. After significant debridement, the tear was clearly identifiable at its distal end. On the left side, the tendon was clearly detached from the tibial bone and folded in on itself underneath the patella. The periosteum was also detached from the bone but remained attached to the ruptured tendons on both sides. The lateral and medial retinacula were detached from their insertions on both tendons.

**Figure 4:**
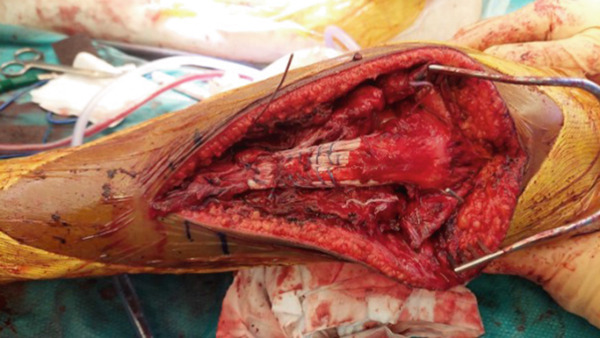
Intraoperative image of the left knee. The patellar tendon has been attached to its insertion point using four anchor sutures, and proximal and distal metallic wires have been passed through the patella and tibia, respectively.

After assessing the correct patellar height, two 3.0‐mm anchor sutures were inserted into each proximal tibia, about 5 mm medial and lateral to the tibial midline, and two lines of running‐type Krackow sutures were applied to reinsert each tendon at the correct tibial height (1 cm below the tibial tubercle) and restore tension. Two supplementary anchor sutures were inserted 1 cm proximal to the first ones and underneath each tendon to attach its distal portion very close to the tibia and to increase the contact surface between the tendon and the bone. Sutures showed good resistance up to 90° of knee flexion. To protect the sutures, a metallic wire frame was inserted through each patella, down to the proximal tibia, just below the tendon insertion point, via a 3.5‐mm cannulated screw previously inserted into the proximal tibia (Figure 4). First, a horizontal hole was created, using a 2.0‐mm drill bit, from the medial to the lateral side through the midsubstance of the patella. A metallic 1.25‐mm wire was then passed through the hole. A second hole was created, using a 2.5‐mm drill bit, on the proximal tibia anterior aspect, about 5–6 cm distal to the articular line and 5–7 mm deep to the anterior cortex. Next, a 3.5‐mm cannulated screw was inserted from the lateral to the medial side into the tibial hole, and the metallic wire was passed through the screw. Using a screw in the proximal tibia prevents the metallic wire from cutting through the bone as tensile forces are applied. The final tension was adjusted by means of two metallic twist ties, one on each side of the frame. Finally, the medial and lateral retinacula were repaired using absorbable sutures.

Augmentation using a metallic wire is intended to protect the anchor sutures during the healing process, especially in cases of bilateral injury, thereby avoiding excessive tension should the patient experience a fall or accidental hyperflexion. This technique is often used in adult repair processes to reduce stress at the reinsertion site [8, 9]. The rationale for our decision to use a metallic wire augmentation for our patient was based on our robust institutional experience with protecting sutures using this technique. Before surgery, potential complications, such as breakage, hardware irritation, stress shielding, patella baja, infection, skin problems, and the need for follow‐up surgery to remove the wires, were all discussed with the patient and her family [10].

**Figure 5:**
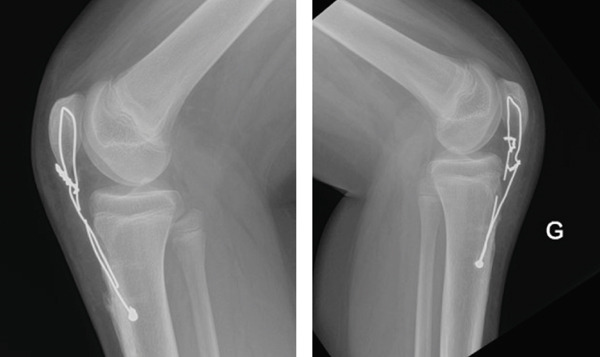
Six‐month, postoperative, right and left lateral knee X‐ray images showing bilateral breakages of the metallic frame and the restoration of normal patellar height.

During the first 2 weeks after surgery, each knee was supported with an extension brace, and weight bearing was only allowed with crutches and braces. Physiotherapy started in Week 3, with simple isometric quadriceps contraction exercises and passive range‐of‐motion (ROM) exercises limited to the knee reaching the 30°–40° angle allowed by a hinged orthopedic brace. A progressive increase in flexion was allowed over the following 4 weeks until the knees reached 90° in Week 6. Rehabilitation continued to improve quadriceps strength and increase ROM. Three months after the operation, the patient had regained bilateral active flexion up to 100° and full quadriceps strength. Anteroposterior and lateral X‐ray images of both knees showed good patellar height (Insall–Salvati ratios: 0.9 left, 0.86 right) with no secondary displacement or ruptures of the metallic wires. Six‐month postsurgical follow‐up revealed full ROM, normal quadriceps strength, and no pain in either knee. The patient was able to walk normally and had restarted gentle running, swimming, and gymnastics classes at school. Six‐month X‐ray images showed the same patellar height in both knees, with bilateral breakage of the metallic frames (Figure 5). The breakage of the metallic wires, some months after surgery, is associated with a good functional outcome and suggests that all the traction forces now pass through the repaired tendon. The metallic wires were subsequently removed from both knees 7 months after the initial operation. The patient was seen again 1 year after surgery (6 months after the wires were removed). Clinical examination at this time revealed full ROM, an absence of pain, and no major complications. The patient reported her full, pain‐free return to sports activities.

## 3. Discussion

An isolated patellar tendon rupture in a young patient is a rare event. Patellar tendon injuries represent just 7% of acute traumatic injuries of the knee extensor mechanism in pediatric populations [7]. A case of bilateral distal patellar tendon rupture, therefore, can be considered almost unique.

To the best of our knowledge, this is only the second case report describing a patient of such a young age (10 years old) sustaining purely tendinous bilateral tears. In 2005, Muratli et al. described bilateral midportion tendon ruptures in a 9‐year‐old boy who subsequently underwent side‐to‐side repair and dual Achilles tendon augmentation [11]. Another similar case was described in a 12‐year‐old who sustained bilateral rupture of the patellar tendons at their proximal insertions on the inferior patellar poles [12]. In 2022, Yonga et al. described a case of bilateral patellar tendon rupture in a 14‐year‐old boy with a history of Osgood–Schlatter disease [13]. He subsequently underwent surgical repair using suture anchors. A few cases have also been described in patients with Ehlers–Danlos syndrome [14] and in patients who had sustained bilateral tibial tubercle bony avulsions [15, 16]. Moreover, these cases occurred in patients with either a collagenopathy (Ehlers–Danlos) or an apophyseal avulsion at the tibial tubercle level. In a study by Yousef and Rosenfeld, five cases of midsubstance patellar tendon rupture were described in older patients, between 12 and 15 years old, two of whom had previously presented with osteogenesis imperfecta [7]. Even in adult populations, bilateral infrapatellar rupture is rare, with only approximately 20 cases reported in the literature [17].

Patellar tendon rupture is rarely spontaneous in young children, but some underlying conditions may make it more likely, including rheumatoid arthritis [18], lupus erythematosus, hyperparathyroidism, corticosteroid use, or long‐term microtrauma such as anterior knee pain and Osgood–Schlatter disease. Surprisingly, our patient presented with none of these conditions; her lesions involved tendinous soft tissues exclusively, with no bony avulsion of the tibial tubercle or the inferior patellar periosteum. Indeed, these lesions seem very unlikely in pediatric populations because of the intrinsic strength of those tendons compared to the apophysis [19].

Although diagnosing bilateral patellar tendon rupture can be challenging [20], the case reported here shows that its early recognition is crucial to achieving a favorable clinical outcome. Timely surgical repair helped to prevent patella alta and a loss of knee extension and strengthened the extensor mechanism. Indeed, not performing surgery could have led to serious gait problems [7]. In our opinion, a lateral knee X‐ray examination constitutes a cornerstone for diagnosis in such cases since it allows clinicians to accurately assess patellar height and search for any bony avulsions on the inferior pole of the patella or the tibial tubercle. However, MRI now also constitutes an essential step in any evaluation of a rupture of the patellar tendon, not only to assess the precise location of the lesion and to determine whether the tear is complete or not [21] but also to identify other serious associated injuries. Indeed, McKinney et al. found a 75% incidence of associated injuries in patients with patellar tendon ruptures, with the most common being tears in the ACL (18%) and the medial meniscus (18%) [22]. Like Otlans et al., we do not consider MRI to be necessary for detecting a patellar tendon rupture (as 99.4% of tears could have been diagnosed clinically [23]), but it is essential for shedding light on any associated lesions. We found no other intra‐articular lesions in the present case, which simplified the surgical treatment.

Different techniques have been described for repairing patellar tendon rupture, such as suture bridges with graft augmentation, transpatellar sutures for proximal avulsions from the inferior patella, transosseous proximal tibial fixation, or anchor sutures for distal avulsions [7]. In our case, for both knees, we chose four 3.0‐mm anchor sutures: two sutures 1 cm distal to the tibial tubercle to reinsert the tendon in a Krackow configuration and two sutures 1 cm proximal to the first ones to attach the tendon tightly onto the tibia over a 1 cm^2^ surface. A transpatellar–transtibial 1.25‐mm metallic wire augmentation was chosen to protect the tendons while they healed. Other augmentation techniques for patellar tendon repair have been described in recent decades, including using autologous gracilis or semitendinous grafts [24], using metallic strand sutures between the bone and the tendon, and even using polyester tape cerclage [8].

The need for augmentation has been documented in cases of frayed tendons, significant gaps, or tendon weakness in adult populations. This is in order to redistribute tensile forces through the tendon and minimize tendon gapping [2]. Unfortunately, given the rarity of the condition in pediatric populations, there is very little literature available on augmentation techniques for patients below 15 years old.

One limitation of this report was the short follow‐up period. Although the surgery’s short‐term outcome was favorable, long‐term follow‐up, out to the patient’s skeletal maturity, could better monitor growth‐related sequelae, such as growth arrest, leg‐length discrepancies, limb deformities, and further tendon ruptures or inflammation.

## 4. Conclusion

Pediatric bilateral patellar tendon rupture represents a serious challenge to health care professionals because of its rarity and intrinsic diagnostic difficulties. Prompt recognition of this genuinely rare problem and early surgical repair, ideally within 7 days of the accident, are crucial to a good clinical outcome for the patient [7].

## Author Contributions

Conceptualization: E.G., O.V., T.G.A., D.C., and G.D.M. Data curation: E.G. Formal analysis: E.G. and G.D.M. Writing—original draft: E.G. Writing—review and editing: E.G., O.V., T.G.A., D.C., and G.D.M.

## Funding

Open access publishing facilitated by Universite de Geneve, as part of the Wiley ‐ Universite de Geneve agreement via the Consortium of Swiss Academic Libraries.

## Disclosure

The authors have nothing to report.

## Ethics Statement

This case report was conducted in accordance with the Declaration of Helsinki and institutional ethical standards. According to institutional regulations, formal ethics committee approval was not required for publication of a single case report, provided that written informed consent was obtained.

## Consent

Written informed consent was obtained from both the patient and her parents for publication of this case report and all accompanying images.

## Conflicts of Interest

The authors declare no conflicts of interest.

## Data Availability

The data that support the findings of this study are available on request from the corresponding author. The data are not publicly available due to privacy or ethical restrictions.

## References

[1] Bentley G. , European Surgical Orthopaedics and Traumatology, The EFORT Textbook, 2014, Springer, Berlin Heidelberg, 10.1007/978-3-642-34746-7.

[2] Brinkman J. C. , Reeson E. , and Chhabra A. , Acute Patellar Tendon Ruptures: An Update on Management, JAAOS: Global Research and Reviews. (2024) 8, no. 4, e24.00060, 10.5435/JAAOSGlobal-D-24-00060, 38569093. PMC10994452 38569093

[3] Matava M. J. , Patellar Tendon Ruptures, Journal of the American Academy of Orthopaedic Surgeons. (1996) 4, no. 6, 287–296, 10.5435/00124635-199611000-00001. 10797196

[4] Hsu H. and Siwiec R. M. , Patellar Tendon Rupture, StatPearls, 2023. 30020647

[5] Dan M. J. , McMahon J. , Parr W. C. H. , Broe D. , Lucas P. , Cross M. , and Walsh W. R. , Evaluation of Intrinsic Biomechanical Risk Factors in Patellar Tendinopathy: A Retrospective Radiographic Case-Control Series, Orthopaedic Journal of Sports Medicine. (2018) 6, no. 12, 2325967118816038, 10.1177/2325967118816038, 2-s2.0-85058245621, 30622997. 30622997 PMC6302276

[6] Steiger C. , Coulin B. , Vendeuvre T. , Tabard-Fougere A. , De Marco G. , Habre C. , Dayer R. , and Ceroni D. , Distal Patellar Tendon Avulsion Associated With an ACL Tear in a Teenager: A Case Report and Review of the Literature, Case Reports in Orthopedics. (2021) 2021, 6686487, 10.1155/2021/6686487, 34327033. 34327033 PMC8302369

[7] Ali Yousef M. A. and Rosenfeld S. , Acute Traumatic Rupture of the Patellar Tendon in Pediatric Population: Case Series and Review of the Literature, Injury. (2017) 48, no. 11, 2515–2521, 10.1016/j.injury.2017.08.069, 2-s2.0-85028746948, 28888715. 28888715

[8] Davis G. , Fellheimer H. S. , McCormick C. , and Freedman K. B. , Repair Techniques for Acute Rupture of the Patellar Tendon: A Systematic Review, Orthopaedic Journal of Sports Medicine. (2026) 14, no. 1, 10.1177/23259671251399844, 41522459. PMC12789414 41522459

[9] Jaramillo Quiceno G. A. , Sarmiento Riveros P. A. , Arias Perez R. D. , Soto Gomez M. P. , and Ramirez A. O. , Augmentation in the Repair of Traumatic Patellar Tendon ruptures. A novel mechanical and biological construct: Technical note, Journal of ISAKOS. (2023) 8, no. 2, 122–127, 10.1016/j.jisako.2022.10.003, 36328345. 36328345

[10] Tandogan R. N. , Terzi E. , Gomez-Barrena E. , Violante B. , and Kayaalp A. , Extensor Mechanism Ruptures, EFORT Open Reviews. (2022) 7, no. 6, 384–395, 10.1530/EOR-22-0021, 35638613. 35638613 PMC9257728

[11] Muratli H. H. , Çelebi L. , Hapa O. , and Biçimoğlu A. , Bilateral Patellar Tendon Rupture in a Child: A Case Report, Knee Surgery, Sports Traumatology, Arthroscopy. (2005) 13, no. 8, 677–682, 10.1007/s00167-005-0620-2, 2-s2.0-27744531602. 15924247

[12] Kim J. R. , Park H. , Roh S. G. , and Shin S. J. , Concurrent Bilateral Patellar Tendon Rupture in a Preadolescent Athlete: A Case Report and Review of the Literature, Journal of Pediatric Orthopaedics B. (2010) 19, no. 6, 511–514, 10.1097/BPB.0b013e32833cb7a0, 2-s2.0-77958154297. 20595921

[13] Yonga O. , Memisoglu K. , Guven M. , Kadioglu B. , and Akman B. , Bilateral Patellar Tendon Rupture in an Adolescent Patient With the Presence of Osgood-Schlatter Disease: A Case Report and Review of the Literature, Annals of Ortopaedics, Trauma And Rehabilitation. (2022) 4, no. 2, https://scientificliterature.org/Orthopaedics/Orthopaedics-22-140.pdf.

[14] Franco H. and Fraser D. , Spontaneous Bilateral Patellar Tendon Rupture in Patient With Ehlers-Danlos Syndrome: A Case Report, Journal of Orthopaedic Case Reports. (2024) 14, no. 10, 124–129, 10.13107/jocr.2024.v14.i10.4834, 39381280. PMC11458247 39381280

[15] Dove J. H. , Perez G. M. , Boulos A. , and Eberson C. P. , Bilateral Tibial Tubercle Avulsion Fractures With an Associated Patellar Tendon Avulsion in an Adolescent Patient, JAAOS Global Research & Reviews. (2023) 7, no. 9, e22-00105, 10.5435/JAAOSGlobal-D-22-00105, 37713638. PMC10508552 37713638

[16] Carbonell-Rosell C. , Pujol O. , Gargallo A. , Lakhani K. , Sevil R. , and Pacha D. , Bilateral Fracture of Anterior Tibial Tuberosity With Complete Patellar Tendon Rupture in an Adolescent Patient, Acta Orthopaedica et Traumatologica Turcica. (2024) 58, no. 4, 247–249, 10.5152/j.aott.2024.23053, 39323265. 39323265 PMC11448756

[17] Noteboom J. T. and Lester M. N. , Bilateral Simultaneous Infrapatellar Tendon Ruptures: A Case Study, Journal of Orthopaedic & Sports Physical Therapy. (1994) 20, no. 3, 166–170, 10.2519/jospt.1994.20.3.166, 2-s2.0-0028060012, 7951294. 7951294

[18] Peiró A. , Ferrandis R. , Garcia L. , and Alcazar E. , Simultaneous and Spontaneous Bilateral Rupture of the Patellar Tendon in Rheumatoid Arthritis. A Case Report, Acta Orthopaedica Scandinavica. (1975) 46, no. 4, 700–703, 10.3109/17453677508989253, 2-s2.0-0016786913, 1180031. 1180031

[19] Kannus P. and Joksa L. , Histopathological Changes Preceding Spontaneous Rupture of a Tendon. A Controlled Study of 891 Patients, Journal of Bone & Joint Surgery. (1991) 73, no. 10, 1507–1525, 10.2106/00004623-199173100-00009, 2-s2.0-0026339892, 1748700. 1748700

[20] Kaneko K. , DeMouy E. H. , Brunet M. E. , and Benzian J. , Radiographic Diagnosis of Quadriceps Tendon Rupture: Analysis of Diagnostic Failure, Journal of Emergency Medicine. (1994) 12, no. 2, 225–229, 10.1016/0736-4679(94)90703-X, 2-s2.0-0028326811, 8207160. 8207160

[21] Weatherall P. T. and Crues J. V. , Musculotendinous Injury, Magnetic Resonance Imaging Clinics of North America. (1995) 3, no. 4, 753–772, 10.1016/S1064-9689(21)00364-0. 8564694

[22] McKinney B. , Cherney S. , and Penna J. , Intra-Articular Knee Injuries in Patients With Knee Extensor Mechanism Ruptures, Knee Surgery, Sports Traumatology, Arthroscopy. (2008) 16, no. 7, 633–638, 10.1007/s00167-008-0516-z, 2-s2.0-46149113685, 18478204. 18478204

[23] Otlans P. , Heimur J. , Sonnier J. H. , Gibby D. , and Freedman K. B. , The Utility of MRI in Evaluating Ruptures of the Patellar Tendon, Orthopaedic Journal of Sports Medicine. (2023) 11, no. 1, 23259671221144980, 10.1177/23259671221144980, 36655018. 36655018 PMC9841853

[24] Espregueira-Mendes J. , Andrade R. , Michael M. J. S. F. , Sarmento A. , Sevivas N. , Rocha R. , and Filho R. B. , Augmentation of Patellar Tendon Repair With Autologous Semitendinosus Graft—Porto Technique, Arthroscopy Techniques. (2017) 6, no. 6, e2271–e2276, 10.1016/j.eats.2017.08.035, 2-s2.0-85035199000, 29349030. 29349030 PMC5765904

